# Pelvic osseous foreign body identified during laparoscopic appendectomy in a child with acute appendicitis: a case report

**DOI:** 10.3389/fmed.2026.1895473

**Published:** 2026-07-06

**Authors:** Han Zhang, Youcheng Zhang, Lingling Pu

**Affiliations:** 1 Department of Pediatric Surgery, Huai'an Maternal and Child Health Care Hospital Affiliated to Yangzhou University, Huai'an, China; 2 Department of Operating Room Nursing, Huai'an Maternal and Child Health Care Hospital Affiliated to Yangzhou University, Huai'an, China

**Keywords:** acute appendicitis, incidental finding, laparoscopic appendectomy, pediatric acute abdomen, pelvic osseous foreign body, peritoneal loose body

## Abstract

**Background:**

Acute appendicitis (AA) is among the most frequent surgical emergencies in children, and laparoscopic appendectomy is widely accepted as the standard treatment. During imaging evaluation for pediatric acute abdomen, incidental pelvic abnormalities may occasionally be identified and can complicate perioperative decision-making. We report a rare case of acute suppurative appendicitis in a child with an unexpected pelvic osseous foreign body detected on preoperative computed tomography (CT).

**Case presentation:**

An 8-year-6-month-old girl presented with a 2-day history of intermittent abdominal pain accompanied by fever. Abdominal CT performed at a local hospital suggested acute appendicitis and additionally revealed a linear hyperdense structure measuring approximately 4.0 cm between the bladder and uterus. Emergency laparoscopic appendectomy was subsequently performed after admission.

**Interventions:**

During laparoscopic exploration, a bone-like foreign body approximately 4.0 cm in length was identified at the pelvic peritoneal reflection without obvious surrounding inflammation or adhesions. Following intraoperative communication with the patient's family and acquisition of informed consent, the foreign body was removed simultaneously. Histopathological examination of the foreign body was declined by the family.

**Outcomes:**

The postoperative course was uneventful, and the patient was discharged on postoperative day 5. Histopathological analysis confirmed acute suppurative appendicitis. At both 1- and 3-month follow-up visits, the patient remained asymptomatic with normal diet, sleep, and bowel function.

**Conclusion:**

This case highlights the importance of careful assessment of incidental findings encountered during the management of pediatric acute abdomen. When intraoperative findings are technically accessible and clinically meaningful, simultaneous management may be considered after adequate evaluation and informed consent.

## Introduction

Acute appendicitis (AA) is the most common surgical cause of acute abdominal pain in children, with the incidence increasing with age and peaking between 10 and 15 years (1, 2). Laparoscopic appendectomy is currently the preferred surgical approach because of its minimal invasiveness, improved visualization, and favorable postoperative recovery (3, 4). With the widespread use of abdominal CT in the evaluation of pediatric acute abdomen, incidental findings unrelated to the primary disease are increasingly encountered. Among these, pelvic hyperdense lesions may occasionally be identified and can present diagnostic uncertainty. Differential diagnoses include phleboliths, calcified mesenteric lymph nodes, myositis ossificans, peritoneal loose bodies (PLBs), and heterotopic ossification (HO) (5–7).

Pelvic osseous foreign bodies are extremely uncommon in children, and the available literature is limited. Most published pediatric cases involve appendicitis secondary to ingested foreign bodies, such as fish bones or other sharp materials, rather than incidentally detected pelvic lesions (6, 8). The etiology of asymptomatic pelvic osseous lesions in children remains unclear. Proposed mechanisms include heterotopic mesenteric ossification (HMO), omental calcification, and sequelae of meconium peritonitis. However, previously reported cases of HMO are usually associated with prior abdominal surgery or significant abdominal trauma, which were absent in our patient (5, 7).

Here, we report the case of an 8-year-old girl with acute suppurative appendicitis in whom a pelvic osseous foreign body was identified on preoperative CT and confirmed during laparoscopic surgery (3, 4). The case highlights the diagnostic considerations and intraoperative management of incidental pelvic findings encountered during pediatric appendectomy.

## Case presentation

### Patient information

An 8-year-and-6-month-old previously healthy girl presented with a 2-day history of intermittent abdominal pain and a half-day history of fever. The abdominal pain began periumbilically and progressively localized to the right lower quadrant. She experienced nausea and 2–3 episodes of non-projectile vomiting containing gastric contents. There was no history of abdominal trauma, prior abdominal surgery, chronic gastrointestinal disease, or blood transfusion. Menarche had not yet occurred. Her parents denied any known history of foreign body ingestion, including fish bones. Family history was unremarkable for hereditary or metabolic disorders.

### Clinical findings

On admission, the patient appeared uncomfortable yet remained alert and hemodynamically stable. Abdominal examination showed diffuse tenderness, most pronounced at McBurney's point in the right lower quadrant, accompanied by mild rebound tenderness and guarding. No palpable abdominal mass or hepatosplenomegaly was noted. Bowel sounds were decreased to approximately 2 per minute. No inguinal abnormalities or skin lesions were observed.

### Diagnostic assessment

Initial laboratory testing revealed leukocytosis with neutrophilia and elevated inflammatory markers (high-sensitivity C-reactive protein and procalcitonin), consistent with acute inflammation. Urinalysis, stool examination, and serum biochemistry were normal.

Ultrasonography was performed as the initial imaging modality at the referring hospital, demonstrating a tubular hypoechoic structure measuring 9 mm in diameter in the right lower quadrant, with loss of mural stratification and absent peristalsis, consistent with appendiceal inflammation. Abdominal CT was subsequently performed at the outside hospital as a complementary study given the atypical concurrent finding of a pelvic hyperdense lesion of uncertain nature that could not be fully characterized by ultrasound alone.

Post-admission repeat CT demonstrated appendiceal enlargement, indistinct margins, and periappendiceal inflammatory changes. An arc-shaped hyperdense lesion measuring approximately 4.0 cm was identified anterior to the uterus and adjacent to the anterior uterine wall, accompanied by mesenteric lymphadenopathy and small pelvic free fluid (Figures 1A–E).

**Figure 1:**
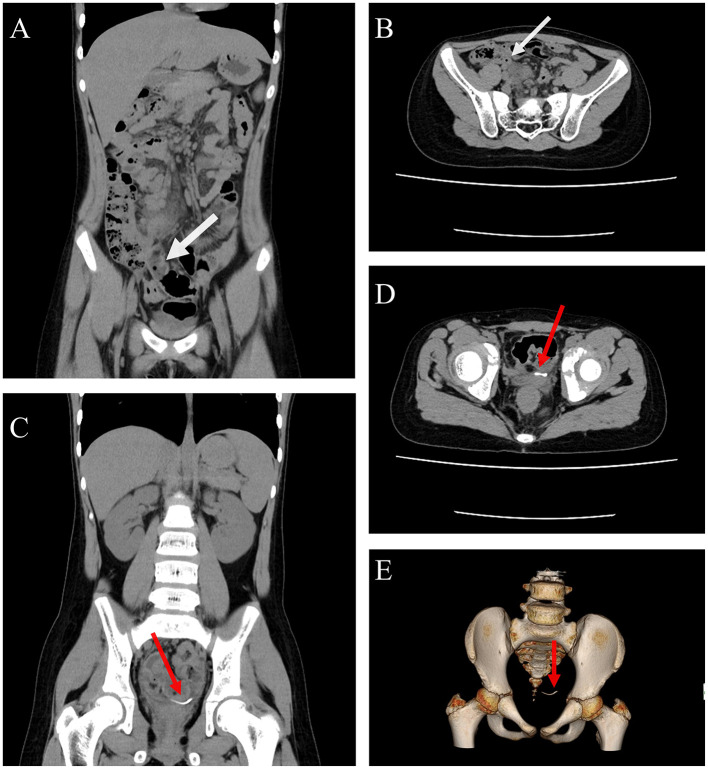
Preoperative abdominal computed tomography (CT) findings of suppurative appendicitis associated with a pelvic bony foreign body. **(A)** Coronal contrast-enhanced abdominal CT image showing an enlarged appendix with surrounding inflammatory changes (white arrow). **(B)** Axial abdominal CT image demonstrating thickening of the appendix and periappendiceal inflammatory infiltration (white arrow). **(C)** Coronal CT image revealing a curved hyperdense foreign body located within the pelvic cavity (red arrow). **(D)** Axial CT image showing the pelvic foreign body adjacent to the rectovesical/rectouterine pouch (red arrow). **(E)** Three-dimensional CT reconstruction clearly demonstrating the shape and location of the pelvic bony foreign body (red arrow).

The preoperative differential diagnosis included Meckel's diverticulitis, intussusception, ovarian torsion, and pelvic inflammatory disease. Given the patient's age, clinical symptoms, laboratory results, and imaging characteristics, the findings were most consistent with acute suppurative appendicitis. The pelvic hyperdense lesion was of uncertain etiology preoperatively; an osseous foreign body could not be excluded. Associated lymphadenopathy and pelvic effusion were attributed to reactive changes secondary to the appendiceal inflammation.

### Therapeutic intervention

Emergency laparoscopic appendectomy was performed under general anesthesia via a standard three-port technique. Intraoperative exploration revealed a markedly swollen and hyperemic appendix (8 cm in length) with purulent exudates on the serosal surface and mild adhesions to adjacent bowel loops. Turbid purulent fluid was present in the pelvis and right paracolic gutter (Figures 2A–C).

**Figure 2:**
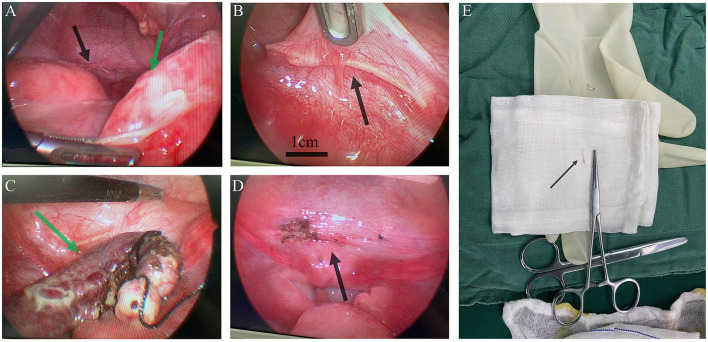
Laparoscopic findings and retrieval of an intrapelvic bony foreign body associated with suppurative appendicitis. **(A)** Intraoperative laparoscopic view demonstrating the suppurative appendix (green arrow) and a bony foreign body embedded near the peritoneal reflection in the pelvic cavity (black arrow). **(B)** Magnified view of the foreign body (black arrow), measuring approximately 4 cm in length. Scale bar = 1 cm. **(C)** Resected suppurative appendix with purulent exudates on the serosal surface (green arrow). **(D)** Operative field after removal of the foreign body, showing the defect at the peritoneal reflection (black arrow). **(E)** Extracted bony foreign body (black arrow), characterized by brittleness and poor toughness, with partial fragmentation after removal.

After appendectomy, further laparoscopic inspection identified an arc-shaped, white-gray, hard foreign body at the pelvic peritoneal reflection between the bladder and uterus (Figures 2A, B). The lesion measured 4.0 cm in length and was freely mobile without surrounding inflammation, encapsulation, or dense adhesion.

Following informed consent, the foreign body was removed piecemeal using laparoscopic grasping forceps and an endoscopic specimen retrieval bag because of its fragile nature. The operative field after removal showed a localized defect at the peritoneal reflection (Figure 2D). Gross examination of the extracted specimen demonstrated brittleness and poor toughness, with partial fragmentation following removal (Figure 2E). The abdominal cavity and pelvis were thoroughly irrigated with warm saline, and a pelvic drainage tube was placed. Histopathological examination of the pelvic lesion was not performed, as requested by the family.

### Follow-up and outcomes

Postoperatively, the patient received intravenous fluids, gastrointestinal rest, and antibiotic therapy with cefoperazone-sulbactam combined with metronidazole. Oral intake was gradually resumed after recovery of bowel function. The pelvic drain was removed after drainage decreased.

Inflammatory markers normalized during hospitalization, and the patient was discharged on postoperative day 5 in stable condition. Histopathological examination of the appendix confirmed acute suppurative appendicitis. No pathological evaluation of the pelvic lesion was available because the family declined further analysis.

At 1- and 3-month follow-up visits, the patient remained asymptomatic with normal appetite, sleep, and bowel function. No postoperative complications or recurrent abdominal symptoms were identified.

## Discussion

In children, a pelvic dense shadow on CT requires careful differential diagnosis. Pelvic phleboliths, though common in adults, are rare in children and typically appear as rounded hyperdense foci with central lucency, unlike the linear arc-shaped lesion seen here (9). Fecaliths generally localize to the appendiceal region with pericecal inflammation—findings present in this case but insufficient to explain the separate pelvic lesion (10). Large peritoneal loose bodies (PLBs) may show oval or round calcification on CT but usually reside within the abdominal cavity rather than at the bladder–uterine peritoneal reflection. Heterotopic mesenteric ossification (HMO) is predominantly reported in adults after abdominal surgery or severe trauma, neither of which applied to this patient (7, 11, 12).

The origin of the pelvic osseous foreign body in this case remains uncertain, yet several potential mechanisms deserve consideration. First, transperitoneal migration following an occult gastrointestinal perforation is plausible: an ingested bone fragment may have caused a microscopic or sealed perforation (e.g., in the appendix, ileum, or sigmoid colon), subsequently migrating into the pelvic peritoneal cavity. The absence of acute peritonitis does not exclude a chronic sealed perforation, as reported in fishbone-induced appendiceal perforation (13, 14). Second, HMO remains a theoretical possibility, though all documented pediatric cases have been associated with prior surgery or major trauma — absent in this patient (7, 11, 12). Third, a large PLB with osseous metaplasia could present as a bone-like pelvic mass, although the 4.0 cm size in this case exceeds typical PLB dimensions (usually < 2 cm) (11). Fourth, clinically silent meconium peritonitis may leave residual intraperitoneal calcifications that present incidentally in later childhood (15, 16). Definitive characterization was precluded because histopathological examination was declined by the family.

The foreign body was arc-shaped, freely mobile, and located at the pelvic peritoneal reflection without surrounding inflammation or encapsulation. Its morphological and imaging features were most consistent with a bone-like lesion or a calcified PLB.

Several factors explain the rarity of pelvic osseous foreign bodies in children. The narrow appendiceal lumen makes entry difficult, while strong peristalsis promotes spontaneous passage of ingested material (17, 18). Symptoms of foreign body–related appendicitis overlap with routine appendicitis, particularly when only low-grade inflammation is present (19, 20). Preoperative CT has the highest diagnostic yield, but detectability depends on the object's composition and scan parameters (21, 22). In this case, CT clearly demonstrated the pelvic dense shadow, guiding targeted exploration and enabling simultaneous removal. This contrasts with many literature reports where such objects were discovered only intraoperatively (20, 22).

Foreign body–associated gastrointestinal complications are more common in children (17); representative cases are summarized in Table 1. In the largest pediatric series to date, Fuller et al. (10), Quitadamo et al. (23) reviewed 56 cases from seven institutions, reporting a mean age of 7.7 years and a predominance of toddlers and early school-age children—an age pattern linked to oral exploration and poor recall of ingestion events. Our patient, aged 8 years 6 months, fits this demographic. However, two features distinguish this case from all prior reports: the foreign body was (a) freely mobile in the pelvic peritoneal cavity without any history of ingestion, surgery, or trauma, and (b) etiologically unrelated to the appendiceal inflammation.

**Table 1:** Summary of published pediatric cases of foreign body–associated appendicitis and related conditions (2006–2025).

No	Title (abbreviated)	Year	Patient age	Causative mechanism	Treatment	Unusual feature
1	Ingested foreign body mimicking appendicolith in a child (29)	2006	18-month-old boy	Ingested gravel; imaging mimicked appendicolith	Open ileal resection + ileocolonic anastomosis	Gravel causing sealed ileal perforation; appendix grossly normal
2	Metallic foreign body in the appendix of a 3-year-old boy (30)	2008	3-year-old boy	Coin-like metallic object impacted in appendiceal lumen	Fluoroscopy-assisted laparoscopic appendectomy	Asymptomatic; diagnosis entirely dependent on imaging
3	Meckel's diverticulum perforation by a wood splinter (31)	2013	4-year-old boy	Wooden splinter perforating Meckel's diverticulum	Open exploration + Meckel's diverticulectomy	Presentation closely mimicked appendicitis
4	Down the rabbit hole—considerations for ingested foreign bodies (32)	2019	7-year-old boy (autism spectrum disorder)	Metallic foreign body retained in appendiceal lumen for 10 months	Laparoscopic appendectomy	Special population; prolonged asymptomatic retention
5	Appendicitis-like picture induced by foreign body in a 2-year-old boy (33)	2020	2-year-old boy	5-cm metallic needle-like object impacted in appendiceal lumen	Emergency appendectomy	Appendicitis itself is rare below age 2; atypical presentation
6	Foreign body appendicitis: A case report and literature review (34)	2025	2-year-10-month-old boy	Screw-like metallic object retained in GI tract for 2 months	Laparoscopic appendectomy	Prolonged retention (2 months) before symptom onset
7	An ingested tooth as a bizarre cause of acute appendicitis (8)	2023	14-year-old boy	Tooth (9 mm × 6 mm) impacted at appendiceal base	Emergency laparoscopic appendectomy	First reported case of tooth-induced appendicitis in a child
8	Acute appendicitis induced by bone fragment ingestion (25)	2024	9-year-old boy	Bone fragment impacted in appendiceal lumen	Laparoscopic appendectomy	Bone fragment represents a calcareous/organic foreign body
9	Previous foreign body ingestion in the appendix causing acute appendicitis (35)	2023	14-year-old girl	Multiple prior foreign body ingestions; metallic needle impacted in appendiceal lumen	Appendectomy	Years of asymptomatic interval between ingestion and appendicitis
10	Foreign body appendicitis coexisting with ascariasis in a pediatric patient (24)	2024	8-year-old girl	Metallic needle ingested 7 months prior + Ascaris lumbricoides co-infection	Open appendectomy	Dual etiology: mechanical foreign body + parasitic infection
11	Metallic pin causing appendicitis: a case from Syria (36)	2025	10-year-old girl	Metallic pin (unknown to patient and family)	Appendectomy	Recurrent urinary tract infections initially obscured the true diagnosis
12	Ingested foreign bodies can cause appendicitis and perforation: a multi-institutional case series (10)	2022	56 pediatric cases (mean age 7.7 years; range: 1 day to 18 years)	Multiple types: hair, plant matter, magnets, metallic objects, BB pellets, marbles, bone fragments, metal chain, wooden splinters	Appendectomy (perforation rate 13%)	Multi-center series (7 institutions); hair and plant matter most common

In the published literature, pediatric foreign body appendicitis involves a wide range of materials, including metallic objects (coins, needles, pins), organic items (fish bones, wooden splinters, seeds), and biological remnants (teeth, bone fragments, parasites) (6, 8, 24). In nearly all cases, the object is located within or immediately adjacent to the appendiceal lumen, causing luminal obstruction and subsequent inflammation (10, 25). The present case deviates substantially from this pattern. Intraoperatively, the osseous foreign body was found freely mobile at the bladder–uterine peritoneal reflection, without any contact or inflammatory reaction with adjacent structures. The appendix showed acute suppurative inflammation without foreign material in the lumen or wall. Therefore, the most accurate diagnosis is primary acute suppurative appendicitis with an incidental, etiologically independent pelvic osseous foreign body. Conflating the two processes would distort the pathophysiological understanding and obscure important lessons regarding incidental findings in pediatric surgery.

Preoperative detection of foreign bodies depends on their density and orientation. Metallic items are easily identified, whereas low-density organic materials such as fish bones may escape notice (26). In our patient, the lesion was readily visible on both pre- and post-transfer CT scans, providing critical preoperative information that guided systematic pelvic exploration. This experience underscores the importance of thorough radiological reporting in pediatric acute abdomen — any dense shadow deserves careful documentation regardless of its apparent relationship to the primary diagnosis.

Determining the etiology of an unexpected foreign body has implications for family counseling and recurrence prevention. In this case, the parents firmly denied any history of ingestion, and there was no record of prior surgery, trauma, or predisposing conditions. While HMO and residual meconium peritonitis were considered, neither was supported by clinical or imaging evidence. The family's decision to forgo histopathological examination represents the chief limitation of this report. To our knowledge, this is the first documented pediatric case of a freely mobile pelvic osseous foreign body of completely unexplained origin in the absence of surgical, traumatic, or ingestion history.

Laparoscopic appendectomy provided an excellent platform for managing both conditions simultaneously. Superior visualization permitted systematic exploration and removal of the incidental lesion without additional incisions (22, 27, 28). Although the object was fragile and required piecemeal extraction, retrieval was accomplished safely using an endoscopic retrieval bag. Intraoperative re-consent was obtained from the guardians, consistent with ethical standards for addressing findings beyond the original surgical plan. Perioperative management followed established protocols, and the patient recovered uneventfully.

This case offers several practical lessons for pediatric surgeons: (1) thorough imaging review is essential, as a diagnosis of appendicitis should not overshadow other findings; (2) systematic abdominopelvic exploration is warranted when preoperative imaging flags additional abnormalities; (3) incidental findings warrant flexible, patient-centered decision-making weighing long-term benefit and risk, with appropriate re-consent; and (4) histopathological examination should be pursued whenever feasible to clarify the nature of unusual lesions.

## Conclusion

This case highlights the importance of carefully evaluating incidental pelvic findings encountered during the management of pediatric acute abdomen. Comprehensive imaging review, systematic intraoperative exploration, and individualized surgical decision-making may facilitate safe management of concurrent abnormalities. Histopathological assessment should be pursued whenever feasible to clarify the nature of unusual lesions.

## Data Availability

The raw data supporting the conclusions of this article will be made available by the authors, without undue reservation.
